# Supplementary material for: Interval walking training in type 2 diabetes: A pilot study to evaluate the applicability as exercise therapy
Source: PLoS One. 2023 May 18;18(5):e0285762. doi: 10.1371/journal.pone.0285762 (PMC10194951; doi:10.1371/journal.pone.0285762)
Supplement: S1 File — (DOCX) [file pone.0285762.s002.docx]

**Clinical trial**

**「Usefulness of Interval Walking Training**

**in patients with type 2 diabetes 」**

**Research Protocol**

Principal investigator: Kouhei Kitajima

Shinshu University School of Medicine

Division of Diabetes, Endocrinology and Metabolism

Department of Internal Medicine

March 12th, 2020, 7^th^ edition.

CONTENTS.

[0. Summary 5](#_Toc488224115)

[0.1. Scheme 5](#_Toc488224116)

[0.2. Objective and significance 6](#_Toc488224117)

[0.3. Target 6](#_Toc488224118)

[0.4. Target enrollment and duration of research 6](#_Toc488224119)

[0.5. Research design 6](#_Toc488224120)

[0.6. Assessment item 6](#_Toc488224121)

[0.7. Contact information 6](#_Toc488224122)

[1. Objective and significance 7](#_Toc488224123)

[2. Background and rationale 7](#_Toc488224124)

[3. Summary of test drugs 8](#_Toc488224125)

[4. Criteria for eligibility 8](#_Toc488224126)

[4.1.　 Selection criteria 8](#_Toc488224127)

[4.2.　 Exclusion criteria 8](#_Toc488224128)

[4.3.　 Subjects requiring consent by a surrogate and the reasons for such consent 8](#_Toc488224129)

[5. Research methods](#_Toc488224130)

[5.1.　 Research design 9](#_Toc488224131)

[5.2.　 Research outline 9](#_Toc488224132)

[5.3.　 Method of administration of research drug 9](#_Toc488224133)

[5.4. Provisions for concomitant medications 13](#_Toc488224134)

[5.4.1.　 Drug use prohibited in combination with other drugs](#_Toc488224135)

[5.4.2.　 Concomitant restricted drugs](#_Toc488224136)

[5.4.3.　 Drugs to be used with caution](#_Toc488224137)

[5.5. Regulations for dose reduction and withdrawal 13](#_Toc488224138)

[5.6. Dealing with subjects after the research is completed 13](#_Toc488224139)

[6. Case registration methods 13](#_Toc488224140)

[6.1.　 Case registration 13](#_Toc488224141)

[7. Endpoint 14](#_Toc488224143)

[7.1.　 Primary endpoint 14](#_Toc488224144)

[7.2.　 Secondary endpoints 14](#_Toc488224145)

[7.3.　 Safety endpoints 14](#_Toc488224145)

[8. Observation and examination items 14](#_Toc488224146)

[8.1.　 Screening evaluation items 14](#_Toc488224145)

[8.2.　 Beginning and End Endpoints 14](#_Toc488224145)

[8.3.　 Evaluation items during the study period 15](#_Toc488224145)

[8.4.　 Post-End Assessment Items 15](#_Toc488224145)

[9. Treatment of adverse events 16](#_Toc488224147)

[9.1.　 Definition of adverse events 16](#_Toc488224148)

[9.1.1.　 Definition of adverse events 16](#_Toc488224149)

[9.1.2.　 Definition of Serious adverse events 17](#_Toc488224150)

[9.2.　 Response to subjects when adverse events occur 17](#_Toc488224151)

[9.3.　 Evaluation and reporting of adverse events 17](#_Toc488224152)

[9.4.　 Predicted adverse events 17](#_Toc488224153)

[10. Target number of enrolled cases 18](#_Toc488224154)

[11. Statistical matter 18](#_Toc488224155)

[11.1.　 Basis for setting the target number of cases to be enrolled 18](#_Toc488224156)

[11.2.　 Statistical analysis methods 18](#_Toc488224157)

[11.2.1.　 Summary of analysis subject 18](#_Toc488224159)

[11.2.2.　 Hypothesis testing analysis on primary endpoints 19](#_Toc488224160)

[11.2.3.　 Analysis of secondary endpoints 19](#_Toc488224161)

[11.2.4.　 Hypothesis-exploratory analysis of primary and secondary endpoints 20](#_Toc488224162)

[12. Completion and submission of case report forms 20](#_Toc488224163)

[12.1.　 Forms and submission deadlines 20](#_Toc488224164)

[13. Monitoring 21](#_Toc488224166)

[14. Audit 21](#_Toc488224167)

[15. Ethical matters 21](#_Toc488224168)

[15.1.　 Rules and regulations to be observed 21](#_Toc488224169)

[15.2.　 Informed consent 21](#_Toc488224170)

[15.3.　 Protection of personal data 23](#_Toc488224171)

[16. Records related to the provision of samples and information 24](#_Toc488224172)

[16.1.　 Information from Shinshu University to JTRC 24](#_Toc488224169)

[16.2.　 Information from JTRC to Shinshu University Hospital 24](#_Toc488224169)

[16.3.　 Points to keep in mind when exchanging information 25](#_Toc488224169)

[17. Changes to research protocols 25](#_Toc488224173)

[18. Research costs 25](#_Toc488224174)

[18.1.　 Research funding and conflict of interest 25](#_Toc488224175)

[18.2.　 Cost-sharing of subjects 25](#_Toc488224176)

[18.3.　 Health hazard response and compensation 26](#_Toc488224177)

[19. Research duration and termination/early termination of the research 26](#_Toc488224178)

[19.1.　 Research period 26](#_Toc488224179)

[19.2.　 Completion of research 26](#_Toc488224180)

[19.3.　 Early discontinuation of studies 26](#_Toc488224181)

[20. Methods of storage and use of medical devices and storage periods 26](#_Toc488224182)

[21. Preservation of records 26](#_Toc488224183)

[22. Publication of research and attribution of results 27](#_Toc488224184)

[22.1.　 Register a research plan 27](#_Toc488224185)

[22.2.　 Attribution of results 27](#_Toc488224186)

[23. Research implementation system 27](#_Toc488224187)

[24. References 29](#_Toc488224188)

[25. Appendix](#_Toc488224189)

# 0. Summary

## Scheme

Major eligibility criteria

・Type 2 diabetes aged 20-80 years

・HbA1c 6.5～10.0%

・BMI 20～34kg/m^2^

Judgment of effectiveness, after 20 weeks of the study

Physical fitness test, Imaging test (CT, MRI), Blood glucose variability assessment

Start of the trial (Start of interval walking training)

In principle, visits every 4 weeks, blood and urine tests will be performed.

20週間

Registration

Target number of enrollment cases：70

Period of case enrollment：After the ethics committee recognized～December 31th, 2019

After registration

Physical fitness test, Imaging test (CT, MRI), Blood glucose variability assessment

## Objective and significance

## To evaluate the usefulness of interval walking training (IWT) for patients with type 2 diabetes in terms of improved glycemic control and changes in body composition (changes in body weight, body fat, liver fat, muscle mass, etc.).

## Target

・Type 2 diabetes patients aged 20-80 years

・HbA1c 6.5～10.0%

・BMI 20～34kg/m^2^

## Target enrollment and duration of research

Target number of enrollment cases：70 cases of IWT

Period of case enrollment：From after the ethics committee recognized to December 31th, 2019

Test period：From after the ethics committee recognized to December 31th, 2020

## Research design

Design features：Single-arm intervention studies

Type of control：none

Randomization：none

Type of blinding：none

## Assessment item

## **Primary endpoint**：Changes in HbA1c levels from baseline (at start) to 20 weeks

## **Secondary endpoints**：Liver fat content by MRI, Abdominal visceral fat and thigh muscle mass by CT, Thigh muscle strength using a muscle testing device, BMI, VO2 peak (maximal oxygen uptake), insulin sensitivity (blood C-peptide, IRI), HDL cholesterol, LDL cholesterol, Triglycerides, Systolic blood pressure, Mean blood glucose and blood glucose variability (using of FreeStyle Libre Pro), Urine albumin, Change in drug dose from baseline (at start) to 20 weeks, Frequency of IWT and achievement rate of the target length of IWT (at least 60 minutes/week of fast walking)

### **Safety endpoints**：Adverse events (hypoglycemia and hypotension), Serious adverse events, Survey on locomotor disorders

## Contact information

【Inquiries about test content】

Research Office：Fourth Department of Internal Medicine, Shinshu University School of Medicine

　3-1-1 Asahi, Matsumoto, Nagano-ken, Japan 390-8621

　TEL：+81-263-37-2686，FAX：+81-263-37-2710

# Objective and significance

To evaluate the usefulness of IWT for patients with type 2 diabetes in terms of improved glycemic control and changes in body composition (changes in body weight, body fat, liver fat, muscle mass, etc.).

# 2. Background and rationale

In a survey by the Ministry of Health, Labor and Welfare in Japan, the estimated number of adults with suspected diabetes increased to 10 million in 2016, and diet and exercise therapy are important for prevention and treatment of type 2 diabetes.

It has been reported that exercise therapy in diabetes patients improved insulin resistance in skeletal muscle and also reduced liver fat mass^1^.

It has also been suggested that exercise therapy is effective in improving HbA1c levels in patients with diabetes (Aerobic exercise group showed -0.46% reduction in HbA1c compared to the control group, resistance exercise group showed -0.37% reduction in HbA1c compared to the control group, and aerobic exercise group + resistance exercise group showed -0.96% reduction in HbA1c compared to the control group)^2^.

With alarming of sarcopenia (muscle weakness) in patients with diabetes, exercise therapy is important even for elderly diabetic patients. In reality, the implementation rate of exercise therapy is low. This is due to a variety of reasons, including patient motivation, time constraints, and lack of habit. Further, it is difficult for medical professionals to instruct patients on exercise therapy because there is no well-established method of exercise therapy for patients with diabetes. IWT "(walking at 70% or more of maximum fitness and walking at 40% or less of maximum fitness alternately for 5 to 10 sets/day for 3 minutes each, repeated at least 4 days/week)" is established by the Department of Sports Medical Sciences, Shinshu University Graduate School of Medicine. There is a report that IWT in patients with diabetes resulted in weight loss (-0.7 kg in a general exercise therapy group and -4.2 kg in IWT group) and a decrease in average blood glucose in CGM (+2 mg/dl in a general exercise therapy group and IWT group -12 mg/dl)^3^. One of the reasons for the effectiveness is the high level of adherence. Another reason may be that IWT allows patients to perform both aerobic and resistance exercise at the same time^4^。

However, there are no papers in Japan evaluating the effectiveness of IWT for patients with type 2 diabetes mellitus.

Therefore, we would like to evaluate blood glucose control and changes in body composition in patients with type 2 diabetes by performing IWT.

We would also like to examine the required amount of IWT to achieve the goal of HbA1c less than 7% in patients with diabetes.

# 3. Summary of test drugs

# Not applicable for this study.

# 4. Criteria for eligibility

# Patients who meet all of the following selection criteria and none of the following exclusion criteria will be considered eligible for enrollment.

## 4.1.　 Selection criteria

1. Type 2 diabetes aged 20-80 years at the time of informed consent.
2. HbA1c 6.5～10.0%
3. BMI 20～34kg/m^2^
4. Patients who have given full explanation of their participation in this study and who have given written consent of their own free will.
5. Patients who are considered by their physician to be eligible for exercise therapy.

## 4.2.　 Exclusion criteria

1. Patients diagnosed with pre-diabetic proliferative or proliferative retinopathy.
2. Urinary albuminuria > 300 mg/gCre, or eGFR < 30 mL/min/1.73 m^2^.
3. History of stroke.
4. History of coronary artery disease.
5. Patients considered inappropriate as participants by the principal investigator.

## 4.3. Subjects requiring consent by a surrogate and the reasons for such consent

Not applicable for this study.

# 5. Research methods

## 5.1.　 Research design

Design features：Single-arm interventional study

Type of control：none

Randomization：none

Type of blinding：none

## 5.2.　 Research outline

## The target enrollment number is 70 patients.

## After enrollment, patients will undergo physical fitness measurements, imaging tests (simple CT of the abdomen and the thighs, and simple MRI of the abdomen), and evaluation of blood glucose variability using FreeStyle Libre Pro, followed by 20 weeks of IWT.

## At the end of the 20 weeks, physical fitness tests, imaging tests, and FreeStyle Libre Pro will be performed to compare before and after IWT. Participants will be encouraged to continue IWT as much as possible until he/she completes the study.

## 5.3.　 How to perform an interventional study

## During the pre-observation period, the participants will undergo simple CT of the abdomen and thighs, and simple MRI of the abdomen, physical fitness tests sponsored by the Research Center of the University of Physical Education (hereinafter referred to as JTRC).

## On screening day, the participants will start to put on FreeStyle Libre Pro, take dietary survey, start to be measured for energy consumed in daily life using JD mate. After that, the participants will wear FreeStyle Libre Pro for 2 weeks, and JD mate for 1 week.

## When taking physical fitness tests, the participants will receive explanation of the content of IWT and be explained again how to handle JD mate.

## The participants will see the doctor on the start date of IWT. The target length of IWT is to walk 60 minutes/week for a total of 20 weeks. As a rule, the participants will be visited every 4 weeks during this period.

## At 18 weeks after the start of the study, FreeStyle Libre Pro will be worn for 2 weeks, and diet survey was performed at the same time. The participants will be also measured for energy consumed in daily life by wearing JD mate for one week.

Within 2 months after the completion of IWT, the patients will be required to undergo simple CT of the abdomen and thighs, simple MRI of the abdomen, and physical fitness tests.

< Details of IWT, the physical fitness tests, FreeStyle Libre Pro, and imaging tests are as follows. >

## Physical fitness test:

Physical fitness tests will be performed at the pre- and post-test observation periods. In this test, maximal oxygen uptake (physical fitness) measurements will be made. The items including physical fitness measurements are listed in the table below.

Measurements

【Morphometrics】

Height, weight, blood pressure, abdominal circumference

【physical fitness test】

Maximal oxygen uptake from walking, muscle strength

## Walking speed is gradually increased, and when reaching full strength, maximal oxygen uptake is measured.

## 2 IWT (Interval Walking Training)：

## Maximal oxygen uptake is the maximum amount of oxygen that an individual can be taken in per minute per kilogram of body weight (mg/kg/min). It is used as an indicator of body endurance (physical fitness).

## IWT is an exercise therapy in which fast walking (>70% of maximal oxygen uptake) and slow walking (<40% of maximal oxygen uptake) are performed for 3 minutes each, for a total of 5 sets (30 minutes)/day, at least 4 times a week. In this clinical trial, the target length of fast walking is set at 60 minutes/week. We would like to examine adherence to IWT, and so, except for participants who withdraw their consent, the study will continue even if the actual fast walking time is not enough. Eventually, all remaining participants will be evaluated after completion of the study. We will ask the participants to continue IWT as much as possible after the 20-week period until they have completed all the evaluation.

## During IWT, an accelerometer and barometer called "JD Mate" are used to estimate the amount of exercise and calories burned. When exercising while wearing this machine, the exercise record is tallied and the rhythm of the fast walking is also indicated with an audible sound.

## FreeStyle Libre Pro：

## This is a one of the continuous blood glucose monitors. When a small, round sensor is attached by a healthcare provider to the back of the upper arm, a very fine needle in the center of the sensor continuously measures glucose levels in the tissue interstitial fluid.

Since the retention period of data imported into JD mate is 2 weeks, we will evaluate the pre- and post-test blood glucose trends by wearing on JD mate at a 2-week pre-observation period and a 2-week period from week 18 to 20 of the study.

## Imaging tests：

## Liver fat will be determined by simple abdominal MRI. Visceral fat at the umbilical line and muscle mass at the center of the right thigh will be determined by simple CT. Muscle strength of the lower limbs will be quantified by the exercise function analyzer "zaRitzBM-220", and the "isometric lower limb muscle strength meter" during the physical fitness tests. These will be performed during the pre- and post-test observation periods, and we will evaluate changes in these items before and after the test (during the post-test observation period, we ask the participants to continue their exercise therapy until all tests have been performed).

5.4. Provisions for concomitant medications (concomitant therapy)

## None

## 5.5. Criteria for stopping exercise therapy

1 Severe complications (motility disorders, hypoglycemia, hypotension) occur.

2 If an attending physician judges that continuation of the exercise therapy is difficult.

3 Criteria for discontinuation of study treatment

（1）When the individual subject of the research withdraws his/her consent.

（2）If it becomes impossible to continue the study treatment due to the occurrence of adverse events, etc.

（3）If found to be ineligible case.

（4）If the subject does not come to the hospital due to relocation, etc.

（5）If the study physician determines that the study should be discontinued.

（6）If the study is discontinued.

※ Temporary interruption of study is not considered a discontinuation, and study will be resumed as soon as it can be resumed.

## 5.6. Dealing with subjects after the study is completed

The principal investigator will provide medical care deemed most appropriate for the subjects, taking the results obtained from this study into account. In addition, although the loan of the JD Mate will cease after the completion of this study, if the research subjects wish to continue IWT, they should consult with the office.

# 6. Case registration methods

## 6.1.　 Case registration

Case registration

1) Registration of cases in this study will be done by contacting the Data Center (in the Center for Clinical Research, Shinshu University Hospital). The principal investigator or research assistant (hereafter referred to as "research staff") will complete the "Registration Form" and submit it to the Data Center with the necessary information regarding research subjects for whom written consent has been obtained and who have been determined to be eligible.

2) The Data Center checks the "Registration Form" for completeness and registers the case in the EDC (UHCT ACReSS).

3) The Data Center will send the "Registration Result Notification Form" to the physician in charge.

4) The principal investigator or research staff will initiate the study after completion of case enrollment.

# 7. Endpoint

## 7.1.　 Primary endpoint

## Percentage change in HbA1c at the beginning of the study and at 20 weeks.

## 7.2.　 Secondary endpoints: Liver fat mass by MRI, abdominal visceral fat and thigh muscle mass by CT, thigh muscle strength by EMG, BMI, VO2 peak (maximal oxygen uptake), insulin sensitivity (blood C-peptide, IRI), HDL cholesterol, LDL cholesterol, triglycerides, systolic blood pressure, mean blood glucose and variability (using FreeStyle Libre Pro), urinary albumin, changes in drug dosage from baseline (start) to 20 weeks (end for some items), frequency of IWT and target achievement rate (at least 60 minutes/week of fast walking).

## 7.3. Safety endpoints：Studies on adverse events (collected only for hypoglycemia and hypotension), serious adverse events (SAEs), and locomotor disorders.

# 8. Observation and examination items

8.1.　 Screening evaluation items

・Patient background：age, gender, medical history (hypertension or not, dyslipidemia or not), smoking history, alcohol consumption history, history of exercise habits, family history of diabetes.

・BMI：height, weight.

・Vital signs: systolic and diastolic blood pressure.

8.2.　 Beginning and End Endpoints

・Body composition evaluation: liver fat content by abdominal MRI, abdominal visceral fat content by CT, thigh muscle mass by CT.

・Thigh muscle strength using isometric lower limb muscle testing equipment.

・Physical fitness measurement (using of JD Mate): VO2 peak (maximum oxygen uptake), target level value for training, maximum heart rate.

・Mean blood glucose and blood glucose variability（using of FreeStyle Libre Pro）

・Daily calorie consumption (using of JD Mate): calorie consumption, number of steps, measurement time (minutes)

・Dietary survey

・Concomitant medications: changes from start to end of diabetes medications (insulin, oral medications), hypertension medications, and lipid disorder medications.

・Insulin sensitivity*: blood CPR, IRI

・Blood tests*: HDL-C, LDL-C, triglycerides, BUN, Cre, eGFR, AST, ALT, fasting blood glucose, HbA1c.

・Urinalysis*: Urinary albumin (creatinine equivalent)

・Vital signs*: systolic and diastolic blood pressure.

・Body weight *

　*…Also evaluate at 4, 8, 12, 16, and 18 week visits.

8.3.　 Evaluation items during the study period

・Training (using of JD Mate): calories burned during IWT, number of steps, training time (minutes).

8.4.　 Post-End Assessment Items

・Surveys for adverse events (collected only for hypoglycemia and hypotension), serious adverse events (SAEs), and locomotor disorders.

Observation, Inspection and Reporting Schedule

|  | **screening** | **previous observation** | **Start Date** | **4 w** | **8w** | **12w** | **16w** | **18w** | **20w** | **after the end** | **time of cessation** |
| --- | --- | --- | --- | --- | --- | --- | --- | --- | --- | --- | --- |
|  |  |  | **baseline** | **±2w** | **±2w** | **±2w** | **-2w　+1ｗ** | **±1w** | **±2w** | **The items implemented from 20 weeks to 28 weeks of the start date.** |  |
| Patient Background | ○ |  |  |  |  |  |  |  |  |  |  |
| Body Composition Assessment |  | ○ |  |  |  |  |  |  |  | ○ |  |
| Physical fitness test |  | ○ |  |  |  |  |  |  |  | ○ |  |
| Interval fast walking |  |  |  |  |  | （20ｗ+ Until the end） |  |  |  |  |  |
| Dietary survey | ○ |  |  |  |  |  |  | ○ |  |  |  |
| Calorie consumption | ○(1w) |  |  |  |  |  |  | ○(1w) |  |  |  |
| BMI | ○ |  | ○ |  | ○ | ○ | ○ |  | ○ |  | (Weight only) |
| Insulin sensitivity |  |  | ○ |  |  |  |  |  | ○ |  |  |
| Blood test | ○ |  | ○ | ○ | ○ | ○ | ○ |  | ○ |  |  |
| Urinalysis | ○ |  | ○ |  |  |  |  |  | ○ |  |  |
| Blood pressure | ○ |  | ○ | ○ | ○ | ○ | ○ |  | ○ |  | ○ |
| Blood glucose level variation | ○(2w) |  |  |  |  |  |  | ○  (18~20w) | |  |  |
| concomitant medications |  |  | ○ |  |  |  |  |  | ○ |  | ○ |
| Survey on locomoter disorders |  |  | ○ | ○ | ○ | ○ | ○ | ○ | ○ | ○ | ○ |
| Adverse events |  |  |  |  |  |  |  |  |  | ○ | ○ |

# 9. Management of adverse events

## 9.1.　 Definition of adverse events

### 9.1.1.　 Definition of adverse events

The term "Adverse Event" shall mean any unwanted or unintended injury or illness or its symptoms (including abnormal laboratory values) that occurs to a research subject, whether or not causally related to the research being conducted.

### 9.1.2.　 Definition of Serious adverse events

　 A serious adverse event (SAE) is an adverse event that falls under any of the following categories.

1. Deadly

2. Life-threatening

3. Requires hospitalization or prolonged hospitalization for treatment

4. Permanent or significant disability or dysfunction

5. Inheritance of congenital anomalies in offspring

## 9.2.　 Response to subjects when adverse events occur

When an adverse event is observed, the principal investigator or research assistant will immediately take appropriate measures and note this in the medical record. In addition, if the study treatment is discontinued due to an adverse event, or if treatment for the adverse event becomes necessary, the subject will be informed.

## 9.3.　 Evaluation and reporting of adverse events

When a serious adverse event is recognized, the research assistant will take necessary measures, such as explaining the event to the research subjects, etc., and promptly report the event to the principal investigator.

When the principal investigator recognizes the occurrence of a serious adverse event, he/she will promptly prepare a "Report on Serious Adverse Events (First Report)" (Shinshu University Medical Clinical Research Plan Form 4) and report it to the Dean of the Shinshu University of School of Medicine. The principal investigator should prepare a "Report on Serious Adverse Events (Second Report)" (Shinshu University Clinical Research Plan Form 5) and report it to the Dean of the Shinshu University School of Medicine within 7 days. In addition, the principle investigator will promptly share the information on the occurrence of the adverse event with the research assistants involved in the implementation of the research.

## 9.4.　 Predicted adverse events

The risk of developing exercise-induced locomotor disease, hypoglycemia, and hypotension is expected.

Adverse events reported for resistance exercise and aerobic exercise in 251 patients wity type 2 diabetes between 39 and 70 years of age, are as follows.

Hypoglycemia frequency 3% (no severe hypoglycemia), trauma frequency 8%, locomotor disorders (upper and lower extremity joint pain, back pain) frequency 17%^5)^.

# 10. Target number of enrolled cases: 70 cases

# 11. Statistical matter

The basic policy of the statistical analysis is shown below. The technical details of the statistical analysis will be specified separately in the statistical analysis plan.

## 11.1.　 Basis for setting the target number of cases to be enrolled

Since this study is an exploratory study, the number of cases was calculated based on feasibility.

Among new and follow-up patients with diabetes at our hospital, the number of patients considered to be eligible for the study is about 70 cases/month, of which we estimate that about 35 cases/month would fit the selection criteria and could be enrolled after obtaining consent.

## 11.2.　 Statistical analysis methods

## ・Primary endpoint：HbA1c

## ・Secondary endpoints：Liver fat mass by MRI, abdominal visceral fat and thigh muscle mass by CT, thigh muscle strength by EMG, BMI, VO2 peak (maximal oxygen uptake), insulin sensitivity (blood C-peptide, IRI), HDL cholesterol, LDL cholesterol, triglycerides, systolic blood pressure, mean blood glucose and variability (using FreeStyle Libre Pro), urinary albumin, changes in drug dosage from baseline (start) to 20 weeks, frequency of IWT and target achievement rate (at least 60 minutes/week of fast walking).

## Safety endpoints：Studies on adverse events (collected only for hypoglycemia and hypotension), serious adverse events (SAEs), and musculoskeletal disorders.

11.2.1.　 Overview of Analysis Objectives

1）Full analysis set (FAS): Participant population enrolled and with at least one analysis available, including baseline.

2）Per protocol set (PPS): Among participants corresponding to FAS, participant population with no significant protocol deviations that would affect the efficacy assessment.

### 3）Safety analysis set (SAF): population of participants who have received at least one protocol treatment.

### 11.2.2.　 Analysis on the primary endpoint (analysis population: FAS)

The percent change in HbA1c from baseline (start date) to 20 weeks in subject i is defined as follows. If HbA1c measurements at either or both time points are missing, the rate of change in HbA1c is considered missing.

$${HbA1c rate of Variation}_{i}=\frac{{HbA1c measurements}_{i,baseline}-{HbA1c measurements}_{i,20w}}{{HbA1c measurements}_{i,baseline}}$$

Estimate the mean and 95% confidence interval of the mean for the rate of change in HbA1c.

### 11.2.3.　 Analysis on secondary endpoints (analysis population: FAS)

### 1）Descriptive summary

## Depending on the data type of each variable, appropriate summary statistics, contingency tables, figures, etc. should be used to summarize the data.

## 2) Exploratory data analysis

Statistical modeling is used to examine the quantitative nature of the following relationships (before -> after).

① Interval fast walking → Outcome

② Background/Baseline → Adherence to interval fast walking

For example, when considering glycemic control for ①, the frequency of IWT (a continuous variable such as hours per week) can be regressed against the achievement of the threshold for change in HbA1c (a binary variable of whether it is less than 7%) in a logistic model to estimate the frequency of IWT required to achieve a given percentage of achievement. ② can be expressed as the weight (influence) of each background/baseline factor with respect to appropriately defined adherence by methods such as regression or search trees.

Not limited to these examples, this analysis will be considered on an exploratory basis. Since specific consideration of models may require data review, it is acceptable to document the model identification at the time of reporting the analysis results, not necessarily in advance.

### 11.2.4.　 Analysis on safety endpoints (analysis population: SAS)

We will prepare and evaluate a list of investigations regarding adverse events (hypoglycemia and hypotension will be collected), serious adverse events (SAEs), and locomotor disorders. In addition, frequency counts are also performed as necessary.

# 12. Completion and submission of case report forms

## 12.1.　 Forms and submission deadlines

Case report forms will be submitted by EDC (Electronic Data Capture System). The principal investigator, research assistant, and collaborator in charge will submit data using EDC as the study progresses. If the form is to be completed by someone other than the physician in charge, confirmation from the physician in charge will be obtained.

Reportable submission period

| No. | Type | Reporting Period |
| --- | --- | --- |
| 1 | Registration form | After obtaining consent |
| 2 | Case Report_Patient Background | Within 2 weeks of each scheduled visit |
| 3 | Case Report_Clinical Laboratory Data | Within 2 weeks of each scheduled visit |
| 4 | Case Report_Body Composition Assessment by Imaging Studies | Within 2 weeks of each scheduled visit |
| 5 | Case report_VO2 peak and isometric leg muscle strength measurements | Within 2 weeks of each scheduled visit |
| 6 | Case Report_Training | Within 2 weeks of each scheduled visit |
| 7 | Case Report_Daily Calorie Consumption | Within 2 weeks of each scheduled visit |
| 8 | Case Report_Concomitant medications | Within 2 weeks of each scheduled visit |
| 9 | Case Report_Adverse Events | Within 2 weeks of each scheduled visit |
| 10 | Musculoskeletal Disorders Questionnaire | Within 2 weeks of each scheduled visit |
| 11 | Case report_discontinued | Within 2 weeks of treatment discontinuation or dropout |

# 13. Monitoring

The monitors will follow the monitoring protocol (including the monitoring plan) prepared by the principal investigator to ensure that the human rights, safety and welfare of the subjects are protected, that the study is conducted in compliance with the latest protocol and "Ethical Guidelines for Medical Research Involving Human Subjects", and that the research data obtained are accurate and complete and can be verified against the relevant records such as medical records.

# 14. Audit

Since this study is an exercise therapy study, it is considered to be less invasive and will not be audited.

# 15. Ethical matters

## 15.1.　 Rules and regulations to be observed

　People involved in this research shall comply with the "Declaration of Helsinki of the World Medical Association" and the "Ethical Guidelines for Medical Research Involving Human Subjects".

## 15.2.　 Informed consent

　The person in charge of the research will provide the consent document approved by the University's Ethics Committee, provide sufficient explanation in writing and orally, and obtain the subject's free and voluntary consent in writing.

When information is obtained that may affect the subject's consent, or when changes are made to the protocol that may affect the subject's consent, the person in charge of the research shall promptly inform the subject, confirm the subject's consent to participate in the research, and obtain prior approval from the University's Ethics Committee to revise the consent document and obtain the subject's re-consent.

In addition, the consent document should include the following information.

1. The name of the research and the fact that permission to conduct the research has been obtained from the director of the research institution.
2. Name of the research institution and name of the principal investigator (when research is conducted jointly with other research institutions, including the name of the joint research institution and the name of the principal investigator of the joint research institution).
3. Purpose and Significance of the Study.
4. Methods and period of the research (including the purpose of use of the samples/information obtained from the Research Subjects).
5. Reason for selection as research subject.
6. Burden and anticipated risks and benefits to research subjects.

⑦ That consent to conduct or continue the research may be withdrawn at any time.（If it is difficult to take measures according to the content of withdrawal from the research subject, etc., that fact and the reason）

1. That the research subjects will not be treated disadvantageously by refusing to consent to the conduct or continuation of the research or by withdrawing their consent.
2. Methods of disclosing information on research.
3. A statement that the Research Subjects, etc. may, at their request, obtain or inspect the research protocol and materials on the research methods to the extent that this does not hinder the protection of the personal information of other Research Subjects, etc. or the securing of originality of the research, and the method of obtaining or inspecting such materials.
4. Handling of personal information, etc. (including the method of anonymization).
5. Methods of storage and disposal of samples and information.
6. Sources of funding for the research, etc., conflicts of interest related to the research by the research institution, and conflicts of interest related to the research by the researcher, etc., including personal earnings.
7. Responding to consultations, etc. from research subjects, etc. and other persons concerned.
8. If there is a financial burden or reward for the research subject, etc., that fact and its details.
9. In the case of research involving medical treatment that exceeds normal practice, matters related to other treatment methods, etc.
10. In the case of research involving medical treatment that goes beyond normal medical treatment, actions related to the provision of medical care to research subjects after the research is conducted.
11. Handling of research results (including incidental findings) if there is a possibility that the implementation of the research will yield important findings regarding the health status of the research subject or genetic characteristics that will be passed on to offspring.
12. In the case of research involving invasive procedures, whether or not compensation is provided for health hazards caused by the research, and the details of such compensation.
13. Specimens and information obtained from research subjects may be used for unspecified future research at the time of obtaining consent from research subjects, etc., or may be provided to other research institutions. In addition, contents assumed at the time of consent should be known.

That, on the assumption that the confidentiality of the research subject will be maintained, persons engaged in monitoring and auditing, and the review committee will have access to the samples and information concerning the research subject to the extent necessary.

## 15.3.　 Protection of personal data

When handling samples and information related to the research, a correspondence table will be created with numbers that are unrelated to the subjects' personal information, and sufficient consideration will be given to protecting the confidentiality of the subjects by anonymizing them. The corresponding table will be strictly managed by the personal information manager. However, when providing information to JTRC, the subject's name and other information will be shared with due consideration for the protection of the subject's confidentiality, since the research cannot be completed without sharing the subject's name and other information. When publishing the results of the research, information that could identify the subjects should not be included. In addition, we will not use the samples and information of the subjects obtained in the research for any purpose other than the purpose of the research.

# 16. Records related to the provision of samples and information

In this study, information will be transferred from Shinshu University Hospital to the Research Center of the University of Physical Education (JTRC).

16.1 Information from Shinshu University to JTRC

Based on "Chapter 5/12 Procedures for Obtaining Informed Consent" of the "Ethical Guidelines for Medical Research Involving Human Subjects", the following records shall be prepared and maintained.

➀Name of the organization to which the information is provided：JTRC

（principal investigator：Mayuka Furihata）

②Information to be provided：Subjects List (Anonymized number, name, gender, date of birth)

③Method of provision: Provided in a file on the Web that can be accessed only by specific account-managed persons.。

The name of the research subject and the record that the consent of the research subject has been obtained shall be kept as a consent document for five years after the end of the research.

16.2 Information from JTRC to Shinshu University Hospital

Based on "Chapter 5/12 Procedures for Obtaining Informed Consent" of the "Ethical Guidelines for Medical Research Involving Human Subjects", the following records shall be prepared and maintained.

1. Name of the organization providing the service：JTRC（Health Promotion Coordinator Mayuka Furihata）

In this study, JTRC conducts the necessary tests, so personal information is inevitably included in the exchange of information. Consent to this fact is obtained from the subject, and the information is exchanged with due care in handling personal information.

1. Sample/information to be provided: List of subjects (anonymized number, name, gender, date of birth), Physical fitness (using JD Mate): VO2 peak (maximum oxygen uptake), target level value for training, maximum heart rate, thigh muscle strength, daily calorie consumption (using JD Mate), complete set of JD Mate data on IWT.
2. Method of provision: Hand delivery on CD-ROM
3. History of data acquisition at the provider institution: Newly acquired for this research.

16.3 Points to keep in mind when exchanging information

① At both donors, the required documents, including the research protocol and the research protocol approved by the lead institution, will be kept as records of the provision of samples and information for five years after the completion of the research.

② In this study, JTRC conducts the necessary tests, so personal information is inevitably included in the exchange of information. Consent to this fact is obtained from the subject, and the information is exchanged with due care in handling personal information.

# 17. Changes to research protocols

Any changes or revisions to the research protocol or consent document for this research must be approved in advance by the University's Ethics Committee.

# 18. Research costs

## 18.1.　 Research funding and conflict of interest

This research will be conducted with the scholarship donation from the department to which the principal investigator belongs. In addition, the person in charge of this research shall declare the necessary information to the Shinshu University Conflict of Interest Management Committee for Clinical Research in accordance with the "Shinshu University School of Medicine Ethics Review Application Procedure" and obtain its review and approval.

## 18.2.　 Cost-sharing of subjects

Participation in this study will increase the number of hospital visits and the number of examinations, resulting in an additional cost burden for the subjects. In particular, imaging tests before and after the study will cost a total of 22440 yen (when insurance covers 30%), but measuring changes in body composition due to exercise therapy is meaningful for the subjects to understand their own physical condition.

On the other hand, we will bear some of the subjects' costs (physical fitness measurement and JD mate usage fees, muscle strength measurement costs, diet survey costs, and FreeStyle Libre Pro costs), and we will fully explain these to the subjects before asking them to make a decision on their participation in the study.

## 18.3.　 Health hazard response and compensation

If any health problems occur to the subjects as a result of this study, appropriate measures, such as examination and treatment, will be taken within the subjects' insurance coverage.

# 19. Research duration and termination/early termination

## 19.1.　 Research period

Period of case registration：After recognition by the Ethics Committee～December 31th, 2019

Testing period：After recognition by the Ethics Committee～December 31th, 2020

## 19.2.　 Completion of research

　The study will be terminated when the data fixation of the last enrolled subject is completed, and the principal investigator will promptly submit a study termination report to the Dean of the School of Medicine.

## 19.3.　 Early termination of studies

　The person in charge of the research will consider whether or not to continue the implementation of the research if any of the following items apply.

1. When it is judged to be extremely difficult to reach the planned number of cases due to difficulties in admitting subjects.
2. When the objective of the research is achieved before the planned number of cases or planned duration is reached.
3. When the Ethics Committee of the University has ordered changes to the implementation plan, etc., and it is deemed difficult to accept these changes.

The principal investigator will discontinue the research if the Ethics Committee of the University recommends or directs discontinuation. When the decision to discontinue the research is made, the decision shall be promptly reported in writing to the Dean of the School of Medicine, together with the reason for the discontinuation.

# 20. Methods of storage and use of medical devices and storage periods

none

# 21. Preservation of records

Samples and information (research data, etc.) pertaining to this research will be stored in the Department's lockable freezers and storage facilities under the supervision of the person responsible for their management (principal investigator) for five years after the publication of the paper, etc., and for ten years after the publication of the results of the paper, etc. for information (materials). At the end of the storage period, the samples should be disposed of in sealed containers or incinerated. Data on paper will be disposed using a shredder while anonymized, and electronic data should be completely erased.

# 22. Publication of research and attribution of results

## 22.1.　 Register a research plan

　The study will be registered as a clinical trial in the database of the UMIN Clinical Trial Registration System (http://www.umin.ac.jp/ctr/index-j.htm), updated as necessary according to changes in the study protocol and the progress of the study, and the results of the study will be registered without delay when the study is completed.

## 22.2.　 Attribution of results

　The results of this research shall belong to Shinshu University. The principal investigator shall publish the results of this research through presentations at relevant conferences and papers.

# 23. Research implementation system

　This study will be conducted under the following structure.

【Research Contributors】

○　Shinshu University Medical School Hospital

Diabetes and Endocrinology 　 Assistant Professor　　 Kouhei Kitajima

　　Shinshu University School of Medicine

　　　Fourth Department of Internal Medicine Professor　 Mitsuhisa Komatu

　　Shinshu University School of Medicine

　 Fourth Department of Internal Medicine

Associate Professor Masanori Yamazaki

Shinshu University Medical School Hospital

　Diabetes and Endocrinology　 Assistant Professor 　 Ai Sato

Shinshu University Medical School Hospital

Diabetes and Endocrinology 　 Assistant Professor　 Ako Oiwa

Shinshu University Medical School Hospital

Diabetes and Endocrinology 　 Assistant Professor　 　　 Yosuke Okubo

Shinshu University Medical School Hospital

Fourth Department of Internal Medicine

Assistant Professor　　　 Yusuke Shibata

Shinshu University School of Medicine

　 Fourth Department of Internal Medicine

Assistant Professor　　　 Junichirou Kitahara

Shinshu University Medical School Hospital

Diabetes and Endocrinology 　 medical staff　　 　　 Yukiko Hattori

Shinshu University Medical School Hospital

Diabetes and Endocrinology 　 medical staff Atsuko Kaneko

Shinshu University Medical School Hospital

Diabetes and Endocrinology 　 medical staff Junko Nakamura

Shinshu University Medical School Hospital

Diabetes and Endocrinology 　 medical staff Keiko Sekido

（○ principal investigator）

【Responsible for information management and physical fitness measurement for interval walking training】

Department of Sports Medical Sciences, Shinshu University Graduate School of Medicine

Health Promotion Coordinator　　 　　 Mayuka Furihata

【Advisors in interval walking training】

Department of Sports Medical Sciences, Shinshu University Graduate School of Medicine

Professor　 　 Shizue Masuki

Aging Biology, Shinshu University Graduate School of Medicine

Special Professor　　　 　　 Hiroshi Nose

【Imaging and Inspection Information Manager】

Department of Radiology, Shinshu University School of Medicine

　　　 Professor　　　　　 Yasunari Hujinaga

【Personal Information Manager】

Shinshu University Medical School Hospital

Diabetes and Endocrinology

Physician's assistant Manami Hosokawa

【Research Office】

Shinshu University Medical School Hospital

Diabetes and Endocrinology　 Medical office　+81 - 263 - 37‐2686

【Data Management Implementation Facility】

Center for Clinical Research, Shinshu University Hospital

Data Management Group

【Statistical Analysis Manager】

SRD Corporation

【Monitoring Facility】

　　Center for Clinical Research, Shinshu University Hospital

Monitoring Groups

# 24. References

1) Tamura,Y. et al.「Effects of diet and exercise on muscle and liver intracellular lipid contents and insulin sensitivity in type2 diabetic patients.」.J clin Endocrinal Metab,90(2005):3191-3196.

2) R. D. Reid. Et.al.「Effects of aerobic exercise, resistance exercise or both,on patient-reported health status and well-being in type 2 diabetes mellitus: a randomised trial」Diabetologia (2010) 53:632–640

3) Kristian,K. et al「The effects of Free Living Interval Walking Training on glycemic control,body composition,and physical fitness in Type 2 diabetic patients」.Diabetic Care 36(2013):228-236.

4) Shuichi Handa et.al.「Effects of aerobic exercise, resistance exercise or both,on atient-reported health status and well-being　in type 2 diabetes mellitus: a randomised trial」Eur J Appl Physiol.23 september 2015

5)Ronald J Sigal et.al.「Effects of aerobic training, resistance training, or both,on glycemic control in type 2 diabetes.」Ann Intern Med. 2007;147:357-369.

:
