# Supplementary material for: Interval walking training in type 2 diabetes: A pilot study to evaluate the applicability as exercise therapy
Source: PLoS One. 2023 May 18;18(5):e0285762. doi: 10.1371/journal.pone.0285762 (PMC10194951; doi:10.1371/journal.pone.0285762)
Supplement: S2 File — (DOCX) [file pone.0285762.s003.docx]

**自主臨床研究**

**「2型糖尿病患者におけるインターバル速歩の有用性」**

**研　究　実　施　計　画　書**

研究責任者 信州大学医学部

糖尿病内分泌代謝内科　医員　　北島　浩平

　　　　作成日

2020年3月12日　計画書案　第7版

目次

[0. 概要 5](#_Toc488224115)

[0.1. シェーマ 5](#_Toc488224116)

[0.2. 目的及び意義 6](#_Toc488224117)

[0.3. 対象 6](#_Toc488224118)

[0.4. 目標登録症例数と試験期間 6](#_Toc488224119)

[0.5. 研究デザイン 6](#_Toc488224120)

[0.6. 評価項目 6](#_Toc488224121)

[0.7. 問い合わせ先 6](#_Toc488224122)

[1. 目的及び意義 7](#_Toc488224123)

[2. 背景と根拠 7](#_Toc488224124)

[3. 試験薬の概要 8](#_Toc488224125)

[4. 適格性の基準 8](#_Toc488224126)

[4.1.　選択基準 8](#_Toc488224127)

[4.2.　除外基準 8](#_Toc488224128)

[4.3.　代諾者による同意が必要な被験者とその理由 8](#_Toc488224129)

[5. 研究の方法](#_Toc488224130)

[5.1.　研究デザイン 8](#_Toc488224131)

[5.2.　研究のアウトライン 9](#_Toc488224132)

[5.3.　試験薬の投与方法 9](#_Toc488224133)

[5.4. 併用薬（併用療法）についての規定 11](#_Toc488224134)

[5.4.1.　併用禁止薬（併用禁止療法）](#_Toc488224135)

[5.4.2.　併用制限薬（併用制限療法）](#_Toc488224136)

[5.4.3.　併用注意薬（併用注意療法）](#_Toc488224137)

[5.5. 減量および休薬についての規定 11](#_Toc488224138)

[5.6. 研究終了後の被験者への対応 11](#_Toc488224139)

[6. 症例登録の方法 11](#_Toc488224140)

[6.1.　症例登録 11](#_Toc488224141)

[7. 評価項目（エンドポイント） 12](#_Toc488224143)

[7.1.　主要評価項目 12](#_Toc488224144)

[7.2.　副次的評価項目 12](#_Toc488224145)

[7.3.　 安全性評価項目 12](#_Toc488224145)

[8. 観察・検査項目 12](#_Toc488224146)

[8.1.　 スクリーニング評価項目 12](#_Toc488224145)

[8.2.　 開始時および終了時評価項目 12](#_Toc488224145)

[8.3.　 開始時から終了時までの評価項目 13](#_Toc488224145)

[8.4.　 終了後評価項目 13](#_Toc488224145)

[9. 有害事象の取り扱い 14](#_Toc488224147)

[9.1.　有害事象の定義 14](#_Toc488224148)

[9.1.1.　有害事象の定義 14](#_Toc488224149)

[9.1.2.　重篤な有害事象の定義 14](#_Toc488224150)

[9.2.　有害事象発現時の被験者への対応 14](#_Toc488224151)

[9.3.　有害事象の評価・報告 14](#_Toc488224152)

[9.4.　予測される有害事象 15](#_Toc488224153)

[10. 目標登録症例数 15](#_Toc488224154)

[11. 統計的事項 15](#_Toc488224155)

[11.1.　目標登録症例数の設定根拠 15](#_Toc488224156)

[11.2.　統計解析方法 15](#_Toc488224157)

[11.2.1.　解析対象の概要 15](#_Toc488224159)

[11.2.2.　主要評価項目に関する仮説検証的解析 16](#_Toc488224160)

[11.2.3.　副次評価項目に関する解析 16](#_Toc488224161)

[11.2.4.　主要評価項目及び副次評価項目に関する仮説探索的解析 16](#_Toc488224162)

[12. 症例報告書の記入と提出 17](#_Toc488224163)

[12.1.　様式と提出期限 17](#_Toc488224164)

[13. モニタリング 17](#_Toc488224166)

[14. 監査 17](#_Toc488224167)

[15. 倫理的事項 18](#_Toc488224168)

[15.1.　遵守すべき諸規則 18](#_Toc488224169)

[15.2.　インフォームド・コンセント 18](#_Toc488224170)

[15.3.　個人情報の保護 19](#_Toc488224171)

[16. 試料・情報の提供に関する記録事項 19](#_Toc488224172)

[16.1.　 信州大学からJTRCへの情報の提供 19](#_Toc488224169)

[16.2.　 JTRCから信州大学医学部附属病院への情報の提供 20](#_Toc488224169)

[16.3.　 情報の授受に際しての留意事項 20](#_Toc488224169)

[17. 研究実施計画書等の変更 20](#_Toc488224173)

[18. 研究の費用 20](#_Toc488224174)

[18.1.　研究資金及び利益相反 20](#_Toc488224175)

[18.2.　被験者の費用負担 21](#_Toc488224176)

[18.3.　健康被害への対応と補償 21](#_Toc488224177)

[19. 研究期間と研究の終了・早期中止 21](#_Toc488224178)

[19.1.　研究期間 21](#_Toc488224179)

[19.2.　研究の終了 21](#_Toc488224180)

[19.3.　研究の早期中止 21](#_Toc488224181)

[20. 医療機器等の保存及び使用方法並びに保存期間 21](#_Toc488224182)

[21. 記録の保存 22](#_Toc488224183)

[22. 研究の公表と成果の帰属 22](#_Toc488224184)

[22.1.　研究計画の登録 22](#_Toc488224185)

[22.2.　成果の帰属 22](#_Toc488224186)

[23. 研究実施体制 22](#_Toc488224187)

[24. 参考資料・参考文献 23](#_Toc488224188)

[25. 付録](#_Toc488224189)

# 0. 概要

## シェーマ

主な適格基準

・30～80歳の2型糖尿病患者

・HbA1c 6.5～10.0%

・BMI 20～34kg/m^2^

試験開始(インターバル速歩開始)

原則4週間毎に受診、血液・尿検査施行

20週間

登録後

体力測定、各種画像検査(CT、MRI)、血糖変動評価(リブレpro)

試験20週後　効果判定

体力測定、各種画像検査(CT、MRI)、血糖変動評価(リブレpro)

登録

目標登録症例数：70例

症例登録期間：倫理委員会承認後～2019年12月31日

## 目的及び意義

## 2型糖尿病患者に対し、インターバル速歩の有用性を、血糖コントロール改善、体組成変化(体重、体脂肪、肝脂肪、筋肉量などの変化)などの面から評価する。

## 対象

- ・30～80歳の2型糖尿病患者
- ・HbA1c 6.5～10.0%
- ・BMI 20～34kg/m^2^

## 目標登録症例数と試験期間

目標登録症例数：インターバル速歩70例

　症例登録期間：倫理委員会承認後～2019年12月31日

　試験実施期間：倫理委員会承認後～2020年12月31日

## 研究デザイン

- - - デザインの特徴：単群介入研究
    - 対照の種類：なし
    - ランダム化：なし
    - 盲検化のレベル：なし

## 評価項目

## **主要評価項目**：HbA1cのベースライン（開始時）と20週時の変化率

## **副次評価項目**：MRIによる肝脂肪量、CTによる腹部内臓脂肪・大腿部筋肉量、筋力測定器による大腿部筋力、BMI、VO2 peak(最高酸素摂取量)、インスリン感受性(血中Cペプチド、IRI)、HDLコレステロール、LDLコレステロール、中性脂肪、収縮期血圧、平均血糖および血糖変動幅(リブレpro使用)、尿中アルブミン、薬剤投与量のベースライン（開始時）と20週時（項目によっては終了時）の変化率、インターバル速歩の頻度・目標(速歩実施60分/週以上)達成率

### **安全性評価項目**：有害事象(低血糖、低血圧のみ収集)、重篤な有害事象（SAE）、運動器障害に関する調査

## 問い合わせ先

【試験内容に関する問い合わせ】

研究事務局：信州大学医学部医学科内科第4

　〒390-8621

　長野県松本市旭3-1-1

　TEL：0263-37-2686，FAX：0263-37-2710

# 目的及び意義

2型糖尿病の患者に対し、インターバル速歩の有用性を、血糖コントロール改善、体組成変化(体重、体脂肪、肝脂肪、筋肉量などの変化)などの面から評価する。

# 2. 背景と根拠

厚生労働省の調査にて、2016年には糖尿病が疑われる成人の推計が1000万人に増加しており、食事療法と運動療法が予防・治療の重要な部分を占める。

糖尿病患者に運動療法を行うことで、骨格筋におけるインスリン抵抗性を改善させ、肝脂肪量も低下させたという報告がある1)。

また、運動療法が糖尿病患者におけるHbA1c低下に有効であることも示唆されている(有酸素運動群はControl群と比較し、HbA1c-0.46%低下、レジタンス運動群はControl群と比較し、HbA1c-0.37%低下、有酸素運動群+レジスタンス運動群はControl群と比較し、-HbA1c-0.96%低下) 2)。

糖尿病患者におけるサルコペニア(筋力低下)が警鐘される中、高齢糖尿病患者においても運動療法が重要と言える。しかし実際、運動療法の実施率は低い。

それは患者の意欲や時間的問題、習慣がないなど様々な理由もあるが、糖尿病患者における運動療法に確立した方法がないため、医療者が指導を行うこと自体も難しいからである。

信州大学スポーツ医科学講座より提唱されている「インターバル速歩(最大体力の70%以上の速歩と40%以下のゆっくり歩きを交互に3分間ずつ5～10セット/日、4日/週以上を目標に繰り返す)」を糖尿病患者に行うことで、体重減少(一般的な運動療法群 -0.7kg、インターバル速歩群 -4.2kg)、CGM(持続血糖測定器)における平均血糖減少(一般的な運動療法群 +2mg/dl、インターバル速歩群 -12mg/dl)などが有意に認められた3)。その理由の一つにアドヒアランスが高いことがある。また、インターバル速歩を行うことで、有酸素運動、レジスタンス運動を同時に実施できるという点もあるだろう4)。

しかし本邦では2型糖尿病患者に対するインターバル速歩の有効性を評価した論文を認めない。

そこで2型糖尿病患者を対象にインターバル速歩を行うことで、血糖コントロールや体組成変化などを評価したい。

また、HbA1c7%未満という糖尿病患者における目標を達成するために行うべきインターバル速歩の頻度についても検討したい。

# 3. 試験薬の概要

# 本研究では該当しない

# 4. 適格性の基準

# 以下の選択基準をすべて満たし、さらに除外基準のいずれにも該当しない患者を登録適格例とする。

## 4.1.　選択基準

1. 同意取得時において年齢が30歳以上、80歳以下の2型糖尿病患者
2. HbA1c 6.5～10.0%
3. BMI 20～34kg/m^2^
4. 本研究への参加にあたり十分な説明を受けた後、患者本人の自由意思に基づき文書による同意が得られた患者。
5. 担当医が運動療法可能と判断した患者。

## 4.2.　除外基準

1. 糖尿病増殖前、増殖網膜症と診断されている患者
2. 腎症3期以上
3. 脳卒中既往
4. 冠動脈疾患既往
5. その他、研究責任者が被験者として不適当と判断した患者

## 4.3.　代諾者による同意が必要な被験者とその理由

## 本研究では該当しない

# 5. 研究の方法

## 5.1.　研究デザイン

- - - デザインの特徴：単群介入研究
    - 対照の種類：なし
    - ランダム化：なし
    - 盲検化のレベル：なし

## 5.2.　研究のアウトライン

## 症例登録人数は70人を目標としている。

症例登録後、体力測定、各種画像検査(腹部単純CT、大腿部単純CT、腹部単純MRI)、リブレproによる血糖変動評価を行い、その後20週間インターバル速歩を開始する。

## 20週間終了後に、体力測定、各種画像検査、リブレproを行い試験前後で変化を比較するが、検査を完遂するまでは極力インターバル速歩を継続していただけるよう推奨する。

##

## 5.3.　介入療法の実施方法及び各種検査の実施方法

## まずは前観察期間の間に、腹部・大腿部単純CT、腹部単純MRI、熟年体育大学リサーチセンター(以下JTRC)が主催する体力測定、リブレproを2週間受けていただく。

## スクリーニング日にリブレproを2週間装着、食事調査を記載、1週間日常生活の消費kcalを熟大メイトにより測定する。

## 体力測定を行う際に、インターバル速歩の内容を説明し、熟大メイトの扱い方を改めて説明する。

## 開始日に受診していただき、その日からインターバル速歩を開始する。

速歩 60分/週を目標とし、合計20週間継続する。この間は基本的に4週間毎の受診とする。

## 試験開始後18週目にリブレproを2週間装着。運動療法後の食事調査を記載し、

熟大メイトを2個装着し、日常生活の消費kcalも1週間測定する。

試験終了後2ヶ月以内を終了後観察期間とし、この間に腹部・大腿部単純CT、腹部単純MRI、体力測定を受けていただく。

## 体力測定、インターバル速歩、リブレpro、画像検査の詳細は以下の通りである。

## 体力測定：

## 前観察期間と試験終了後観察期間に施行し、試験前後の最高酸素摂取量(体力)測定を実施する。体力測定時の項目は下記の表の通りである。

**測定項目**

【形態測定】身長、体重、体脂肪率、血圧、腹部周囲径

【体力測定】歩行による最高酸素摂取量測定、筋力測定

## 最高酸素摂取量は、体力測定の際に徐々に歩行速度を挙げていき、全力となった際に測定する。

## インターバル速歩：

## 最高酸素摂取量とは、1分間に体重1kgあたり取り込むことのできる酸素量(mg/kg/分)のことである。これは全身持久力(体力)の指標として用いられる。

## インターバル速歩とは、速歩(最高酸素摂取量の70%以上)と緩歩(最高酸素摂取量の40%以下)を各3分、1setとし、合計5set(30分)/日とし、これを週4回以上行う運動療法である。今回の臨床試験では目標を速歩 60分/週と設定するが、インターバル速歩のアドヒアランスについても研究したいため、あくまで目標と考え、実際の施行時間が増減したり、同意撤回がない限り中止休止があったとしても研究を継続し、終了後の評価を受けていただく。また、20週間終了後も、検査を完遂するまでは極力インターバル速歩を継続していただけるよう推奨する。

## インターバル速歩中は、「熟大メイト」という加速度計・気圧計を用いて運動量・消費カロリーを推算する。この機械を装着して運動を行うと、運動記録を集計し、また速歩のリズムも音で教えてくれる。

## リブレpro：

## 持続血糖測定器の1つ。小さい丸いセンサーを研究対象者の上腕部裏側に医療者が装着することで、センサー中心部の極細の針が組織間質液中のグルコース値を持続的に測定する。

保存可能期間が14日間のため、前観察期間に2週間、試験18週から20週の2週間に施行し、試験前後の血糖推移を評価したい。

## 画像検査：

## 腹部単純MRIで肝脂肪を、単純CTで臍のラインの内臓脂肪と右大腿の中心部における筋肉量を、運動機能分析装置「zaRitzBM-220」と体力測定の際に「等尺性下肢筋力測定器」で下肢の筋力を定量する。これらを前観察期間と試験終了後観察期間に施行し、試験前後の変化を評価したい(試験終了後観察期間は、すべての検査が施行されるまでは運動療法を継続していただくようお願いする)。

5.4. 併用薬（併用療法）についての規定

## 制限なし

## 5.5. 運動療法の中止の基準

①重度な合併症(運動器障害、低血糖、低血圧)が生じる。

②担当医が運動療法継続不可能と判断した場合。

試験治療の中止基準

（1）研究対象者本人の同意撤回があった場合

（2）有害事象の発現等により試験治療継続が不可能となった場合

（3）不適格症例であると判明した場合

（4）転居等により研究対象者が来院しない場合

（5）試験参加医師が中止すべきと判断した場合

（6）試験が中止された場合

※なお、一時的な試験治療の中断は中止とせず、再開可能となれば速やかに試験治療を再開する。

## 5.6. 研究終了後の被験者への対応

この研究で得られた成果も含めて、研究責任者は被験者に対し最も適切と考えられる医療を提供する。また、本研究終了後はインターバル速歩と熟大メイトの貸し出しは終了となるが、もし研究対象者が継続を希望された場合は、事務局にご相談いただく。

# 6. 症例登録の方法

## 6.1.　症例登録

症例登録

1) 本研究での症例登録は、データセンター（信州大学医学部附属病院臨床研究支援センター内）への連絡による。研究責任者又は研究分担者（以下、研究担当者）は文書同意が得られ、適格と判定した研究対象者に関する必要事項を、「登録票」に記入し、データセンター(臨床研究支援センター)へ提出する。

2) データセンターは、「登録票」の記載内容に不備がないかを確認し、EDC（UHCT ACReSS）に症例を登録する。

3) データセンターは、「登録結果通知書」を担当医師に送付する。

4) 研究責任者又は研究分担者は、症例登録の完了後、本研究を開始する。

# 7. 評価項目（エンドポイント）

## 7.1.　主要評価項目

## HbA1cの試験開始時と20週時の変化率

## 7.2.　副次的評価項目：MRIによる肝脂肪量、CTによる腹部内臓脂肪・大腿部筋肉量、筋力測定器による大腿部筋力、BMI、VO2 peak(最高酸素摂取量)、インスリン感受性(血中Cペプチド、IRI)、HDLコレステロール、LDLコレステロール、中性脂肪、収縮期血圧、平均血糖および血糖変動幅(リブレpro使用)、尿中アルブミン、薬剤投与量のベースライン（開始時）と20週時（項目によっては終了時）の変化率、インターバル速歩の頻度・目標(速歩実施60分/週以上)達成率

## 7.3. 安全性評価項目：有害事象(低血糖、低血圧のみ収集)、重篤な有害事象（SAE）、運動器障害に関する調査

#

# 8. 観察・検査項目

8.1.　スクリーニング評価項目

・患者背景：年齢、性別、既往歴(高血圧症有無、脂質異常症有無)、喫煙歴、飲酒歴、元の運動習慣、糖尿病の家族歴

・BMI：身長、体重

・バイタルサイン：収縮期血圧、拡張期血圧

8.2.　開始時および終了時評価項目

・体組成評価：腹部MRIによる肝脂肪量、CTによる腹部内臓脂肪量、CTによる大腿部筋肉量

・等尺性下肢筋力測定器による大腿部筋力量

・体力測定(熟大メイト)：VO2 peak(最高酸素摂取量)、トレーニングの目標レベル値、最大心拍数

・平均血糖および血糖変動幅（リブレpro使用）

・日常消費カロリー(熟大メイト使用) ：消費カロリー、歩数、測定時間(分)

・食事調査

・併用薬：糖尿病治療薬(インスリン、内服薬)、高血圧治療薬、脂質異常治療薬の開始時から終了時までの変化

・インスリン感受性*：血中CPR、IRI

・血液検査*：HDL-C、LDL-C、中性脂肪、BUN、Cre、eGFR、AST、ALT、空腹時血糖、HbA1c

・尿検査*：尿中アルブミン(クレアチニン換算)

・バイタルサイン*：収縮期血圧、拡張期血圧

・体重*

　*…4週時、8週時、12週時、16週時、18週時の来院時にも評価を行う。

8.3.　開始時から終了時までの評価項目

・トレーニング(熟大メイト使用)：体力測定時消費カロリー、歩数、トレーニング時間(分)

8.4.　終了後評価項目

・有害事象(低血糖、低血圧のみ収集)、重篤な有害事象（SAE）、運動器障害に関する調査

観察・検査・報告スケジュール

|  | **ｽｸﾘｰ**  **ﾆﾝｸﾞ** | **前観察** | **開始日** | **4週** | **8週** | **12週** | **16週** | **18週** | **20週** | **終了後** | **中止時** |
| --- | --- | --- | --- | --- | --- | --- | --- | --- | --- | --- | --- |
|  |  |  | **ベースライン** | **±2w** | **±2w** | **±2w** | **-2w　+1ｗ** | **±1w** | **±2w** | **開始日20週から28週までに実施** |  |
| 患者背景 | ○ |  |  |  |  |  |  |  |  |  |  |
| 体組成評価 |  | ○ |  |  |  |  |  |  |  | ○ |  |
| 体力測定 |  | ○ |  |  |  |  |  |  |  | ○ |  |
| インターバル速歩 |  |  |  |  |  |  | （20週+終了時まで） |  |  |  |  |
| 食事調査 | ○ |  |  |  |  |  |  | ○ |  |  |  |
| 消費カロリー | ○(1w) |  |  |  |  |  |  | ○(1w) |  |  |  |
| BMI | ○ |  | ○ |  | ○ | ○ | ○ |  | ○ |  | (体重のみ) |
| インスリン感受性 |  |  | ○ |  |  |  |  |  | ○ |  |  |
| 血液検査 | ○ |  | ○ | ○ | ○ | ○ | ○ |  | ○ |  |  |
| 尿検査 | ○ |  | ○ |  |  |  |  |  | ○ |  |  |
| 血圧 | ○ |  | ○ | ○ | ○ | ○ | ○ |  | ○ |  | ○ |
| 血糖変動 | ○(2w) |  |  |  |  |  |  | ○  (18~20w) | |  |  |
| 併用薬 |  |  | ○ |  |  |  |  |  | ○ |  | ○ |
| 運動器障害に関する調査 |  |  | ○ | ○ | ○ | ○ | ○ | ○ | ○ | ○ | ○ |
| 有害事象 |  |  |  |  |  |  |  |  |  | ○ | ○ |

# 9. 有害事象の取り扱い

## 9.1.　有害事象の定義

### 9.1.1.　有害事象の定義

有害事象とは、実施された研究との因果関係の有無を問わず、研究対象者に生じた全ての好ましくない又は意図しない傷病もしくはその徴候（臨床検査値の異常を含む。）とする。

### 9.1.2.　重篤な有害事象の定義

　 重篤な有害事象（SAE：Severe Adverse Event）とは、有害事象のうち次に掲げるいずれかに該当するものをいう。

1.死に至るもの

2.生命を脅かすもの

3.治療のための入院又は入院期間の延長が必要となるもの

4.永続的又は顕著な障害・機能不全に陥るもの

5.子孫に先天異常を来すもの

## 9.2.　有害事象発現時の被験者への対応

研究責任者または研究分担者は、有害事象を認めたときは、直ちに適切な処置を行うとともに、診療録にその旨を記載する。また、有害事象によって試験治療を中止した場合や、有害事象に対する治療が必要となった場合には、被験者にその旨を伝える。

## 9.3.　有害事象の評価・報告

研究担当者は、重篤な有害事象の発生を知った場合には、研究対象者等への説明等、必要な措置を講じるとともに、速やかに研究責任者に報告する。

研究責任者は、重篤な有害事象の発生を知った場合には、速やかに、「重篤な有害事象に関する報告書（第一報）」（信大医臨床研究計画様式 4）を作成し、医学部長に報告する。研究責任者は、原則として 7 日以内に、「重篤な有害事象に関する報告書（第二報）」（信大 医臨床研究計画様式 5）を作成し、医学部長に報告する。また、速やかに当該研究の実施に携わる研究担当者等に対して、当該有害事象の発生に係る情報を共有する

## 9.4.　予測される有害事象

運動による運動器疾患の発症、低血糖、低血圧発症のリスクが予想される。

これまでにレジスタンス運動と有酸素運動を、39～70歳までの251人の2型糖尿病患者を対象に実施した際に報告された有害事象は以下の通り。

低血糖頻度 3%(重度低血糖はなし)、外傷頻度 8%、運動器疾患(上下肢関節痛、腰痛)頻度 17% ^5)^

# 10. 目標登録症例数

インターバル速歩　 70例

11. 統計的事項

統計解析の基本的な方針を下記に示す。実施に関しての技術的詳細等は統計解析計画書にて別途定める。

## 11.1.　目標登録症例数の設定根拠

本研究は探索的研究であるため、実施可能性から症例数を算出した。

当院の糖尿病の新規および経過観察患者のうち、当該研究の対象と考えられる症例数は70例／月程度であり、このうち、当該選択基準に当てはまり、同意を取得して登録可能である症例は約35例/月程度と見積もり、登録期間実質約2か月より、症例数を70例とした。

## 11.2.　解析項目・方法

## ・主要評価項目：HbA1c

## ・副次評価項目：MRIによる肝脂肪量、CTによる腹部内臓脂肪・大腿部筋肉量、筋力測定器による大腿部筋力、BMI、VO2 peak(最高酸素摂取量)、インスリン感受性(血中Cペプチド、IRI)、HDLコレステロール、LDLコレステロール、中性脂肪、収縮期血圧、平均血糖および血糖変動幅(リブレpro使用)、尿中アルブミン、薬剤投与量のベースライン（開始時）と20週時（項目によっては終了時）の変化率、インターバル速歩の頻度・目標(速歩実施60分/週以上)達成率

## **安全性評価項目**：有害事象(低血糖、低血圧のみ収集)、重篤な有害事象（SAE）、運動器障害に関する調査

11.2.1.　解析対象の概要

1）主要な解析対象集団（Full analysis set: FAS）：登録され、ベースラインを含む少なくとも1回以上の解析可能な結果を得られている患者集団

2）プロトコールに従った集団（Per protocol set: PPS）：FAS のうち有効性評価に影響を及ぼすような重大なプロトコール逸脱がなかった患者

### 3）安全性解析対象集団（Safety analysis set: SAF）：プロトコール治療を少なくとも一度受けた患者集団

### 11.2.2.　主要評価項目に関する解析（解析対象集団：FAS）

被験者 $i$ におけるベースライン（開始日）および20週のHbA1cの変化率を次のように定義する。いずれか又は両方の時点におけるHbA1c測定値が欠損した場合は、HbA1c変化率は欠損とする。

$${HbA1c変化率}_{i}=\frac{{HbA1c測定値}_{i,ベースライン}-{HbA1c測定値}_{i,20週}}{{HbA1c測定値}_{i,ベースライン}}$$

HbA1c変化率の平均値及び平均値の95％信頼区間を推定する。

### 11.2.3.　副次評価項目に関する解析（解析対象集団：FAS）

## 1）記述的要約

## 各変数のデータ型に応じて、要約統計量、分割表、図等を用いて適切な要約を行う。

## 2) 探索的解析

次のような関係（前→後）の定量性について、統計モデリングにより検討する。

① インターバル速歩 → アウトカム

② 背景/ベースライン → インターバル速歩のアドヒランス

例えば①について血糖コントロールを考える場合、インターバル歩行の頻度（週当たりの実施時間などの連続型変数）に対して、HbA1cの変化閾値の達成（7%未満となったか否かの2値変数）をロジスティックモデルで回帰させることができ、所定の達成割合を得るために必要なインターバル歩行の頻度を推定することができる。②については、回帰あるいは探索木などの手法により、適当に定義したアドヒランスに対する、各背景/ベースライン因子の重み（影響度）として表現することなどができる。

これらの例に限定せず、本解析は探索的に検討することとする。モデルの具体的検討に際しては、データレビューを要する場合もあるため、モデルの特定については必ずしも事前でなく、解析結果報告時に文書化することも許容する。

### 11.2.4.　安全性評価項目に関する解析（解析対象集団：SAS）

有害事象(低血糖、低血圧のみ収集)、重篤な有害事象（SAE）、運動器障害に関する調査について、一覧表を作成して評価する。また、必要に応じて頻度集計による要約も行う。

# 12. 症例報告書の記入と提出

## 12.1.　様式と提出期限

症例報告書の提出は、EDC（電子データ採取システム）によって行う。研究責任者および研究分担者、担当協力者は、試験の進捗にあわせてEDCを用いてデータ提出を行う。担当医師以外が記入する場合は担当医師の確認を得る。

報告対象の提出時期

| No. | 種類 | 報告時期 |
| --- | --- | --- |
| 1 | 登録票 | 同意取得後 |
| 2 | 症例報告書_患者背景 | 規定の各来院後2週間以内 |
| 3 | 症例報告書_臨床検査値 | 規定の各来院後2週間以内 |
| 4 | 症例報告書_画像検査による体組成評価 | 規定の各来院後2週間以内 |
| 5 | 症例報告書_VO2 peakおよび等尺性下肢筋力測定 | 規定の各来院後2週間以内 |
| 6 | 症例報告書_トレーニング | 規定の来院後2週間以内 |
| 7 | 症例報告書_日常消費カロリー | 規定の各来院後2週間以内 |
| 8 | 症例報告書_併用薬 | 規定の来院後2週間以内 |
| 9 | 症例報告書_有害事象 | 規定の来院後2週間以内 |
| 10 | 運動器障害アンケート | 規定の来院後2週間以内 |
| 11 | 症例報告書_中止 | 治療中止または脱落後2週間以内 |

# 13. モニタリング

モニターは研究責任者が作成したモニタリングに関する手順書（モニタリング計画を含む）に従い、被験者の人権、安全性及び福祉が保護されていること、本研究が最新のプロトコール、「人を対象とする医学系研究に関する倫理指針」を遵守して実施されていること及び得られた研究データなどが正確かつ完全で、診療録等の関連記録に照らして検証できることを確認する。

# 14. 監査

本研究は運動療法を行う研究であることから、侵襲性は少ないと考えられ、監査は実施しない。

# 15. 倫理的事項

## 15.1.　遵守すべき諸規則

　本研究の関係者は「世界医師会ヘルシンキ宣言」および「人を対象とする医学系研究に関する倫理指針」を遵守する。

## 15.2.　インフォームド・コンセント

　研究担当者は、本学倫理委員会で承認の得られた同意説明文書を渡し、文書および口頭による十分な説明を行い、被験者の自由意思による同意を文書で取得する。

研究担当者は、被験者の同意に影響を及ぼす情報が得られたときや、被験者の同意に影響を及ぼすような実施計画等の変更が行われるときは、速やかに被験者に情報提供し、研究に参加するか否かについて被験者の意思を予め確認するとともに、事前に本学倫理委員会の承認を得て同意説明文書等の改訂を行い、被験者の再同意を得ることとする。

なお、同意説明文書は、以下の内容を含むものとする。

1. 研究の名称及び当該研究の実施について研究機関の長の許可を受けている旨
2. 研究機関の名称及び研究責任者の氏名（他の研究機関と共同して研究を実施する場合には、共同研究機関の名称及び共同研究機関の研究責任者の氏名を含む。
3. 研究の目的及び意義
4. 研究の方法（研究対象者から取得された試料・情報の利用目的を含む。）及び期間
5. 研究対象者として選定された理由
6. 研究対象者に生じる負担並びに予測されるリスク及び利益

⑦研究が実施又は継続されることに同意した場合であっても随時これを撤回できる旨（研究対象者等からの撤回の内容に従った措置を講じることが困難となる場合があるときは、その旨及びその理由）

⑧研究が実施又は継続されることに同意しないこと又は同意を撤回することによって　研究対象者等が不利益な取扱いを受けない旨

1. 研究に関する情報公開の方法

⑩研究対象者等の求めに応じて、他の研究対象者等の個人情報等の保護及び当該研究の独創性の確保に支障がない範囲内で研究計画書及び研究の方法に関する資料を入　手又は閲覧できる旨並びにその入手又は閲覧の方法

1. 個人情報等の取扱い（匿名化する場合にはその方法を含む。）
2. 試料・情報の保管及び廃棄の方法

⑬研究の資金源等、研究機関の研究に係る利益相反及び個人の収益等、研究者等の研究に係る利益相反に関する状況

1. 研究対象者等及びその関係者からの相談等への対応
2. 研究対象者等に経済的負担又は謝礼がある場合には、その旨及びその内容
3. 通常の診療を超える医療行為を伴う研究の場合には、他の治療方法等に関する事項

⑰通常の診療を超える医療行為を伴う研究の場合には、研究対象者への研究実施後における医療の提供に関する対応

⑱研究の実施に伴い、研究対象者の健康、子孫に受け継がれ得る遺伝的特徴等に関する重要な知見が得られる可能性がある場合には、研究対象者に係る研究結果（偶発的所見を含む。）の取扱い

⑲侵襲を伴う研究の場合には、当該研究によって生じた健康被害に対する補償の有無　及びその内容

⑳研究対象者から取得された試料・情報について、研究対象者等から同意を受ける時点では特定されない将来の研究のために用いられる可能性又は他の研究機関に提供する可能性がある場合には、その旨と同意を受ける時点において想定される内容

研究対象者の秘密が保全されることを前提として、モニタリングに従事する者及び監査に従事する者並びに審査委員会が、必要な範囲内において当該研究対象者に関する試料・情報を閲覧する旨

## 15.3.　個人情報の保護

研究実施に係る試料・情報を取扱う際は、被験者の個人情報とは無関係の番号を付して、対応表を作成し、匿名化を行い被験者の秘密保護に十分配慮する。対応表は個人情報管理者が厳重に管理するが、JTRCへ情報を提供する際は被験者の名前等を共有しなければ研究が成り立たないので、被験者の秘密保護に十分配慮して共有する。研究の結果を公表する際は、被験者を特定できる情報を含まないようにする。また、研究の目的以外に、研究で得られた被験者の試料・情報を使用しない。

# 16. 試料・情報の提供に関する記録事項

本研究においては信州大学医学部附属病院より熟年体育大学リサーチセンター(JTRC)との情報の授受を行う。

16.1 信州大学からJTRCへの情報の提供

「人を対象とする医学系研究に関する倫理指針」の「第5章／第12 インフォームド・コンセントを受ける手続等」に基づき、下記の事項の記録を作成・保管する。

1. 提供先の機関名：JTRC　（研究責任者：降幡　真由佳）

②提供を行う情報：被験者一覧(匿名化番号、氏名、性別、生年月日)

③提供方法：アカウント管理された特定の者のみがアクセスできるWeb上のファイルで提供する。

研究対象者の氏名等、及び研究対象者等の同意を受けている旨に関する記録として、同意文書を研究終了後５年間保管するものとする。

16.2 JTRCから信州大学医学部附属病院への情報の提供

「人を対象とする医学系研究に関する倫理指針」の「第5章／第12 インフォームド・コンセントを受ける手続等」に基づき、下記の事項の記録を作成・保管する。

1. 提供元の機関名：JTRC（健康推進コーディネータ 降幡 真由佳）

本研究では必要な検査をJTRCにて実施するため、情報の授受にあたり、個人情報を含まざるを得ない。この旨を被験者に同意を得て、個人情報の取り扱いには十分に注意をして、情報の授受を行う。

1. 提供を受ける試料・情報：被験者一覧(匿名化番号、氏名、性別、生年月日)、体力測定(熟大メイト)：VO2 peak(最高酸素摂取量)、トレーニングの目標レベル値、最大心拍数、大腿部筋力量、日常消費カロリー(熟大メイト使用)、インターバル速歩に関する熟大メイトのデータ一式
2. 提供方法：CD-ROMにて手渡し

④提供元の機関における取得の経緯：本研究の為に新たに取得

16.3情報の授受に際しての留意事項

①両提供元において、試料・情報の提供に関する記録として、本研究実施計画書及び主任施設で承認された研究実施計画書等、必要事項を記載した文書を研究終了後５年間保管する。

②本研究では必要な検査を熟年体育大学リサーチセンターにて、実施するため、情報の授受にあたり、個人情報を含まざるを得ない。この旨を被験者に説明し同意を得て、情報の授受を行う。

# 17. 研究実施計画書等の変更

本研究の研究実施計画書や同意説明文書の変更または改訂を行う場合は、あらかじめ本学倫理委員会の承認を得る。

# 18. 研究の費用

## 18.1.　研究資金及び利益相反

本研究は、研究責任者が所属する教室の奨学寄附金で実施する。また、本研究の研究担当者は、「信州大学医学部倫理審査申請の手順」にしたがって、信州大学臨床研究に係る利益相反マネジメント委員会に必要事項を申告し、その審査と承認を得るものとする。

## 18.2.　被験者の費用負担

本研究への参加により、通院回数および検査数が増加するため、被験者への費用負担が発生する。特に画像検査は試験前後で合計22440円(保険負担3割の場合)負担費用が発生するが、運動療法による体組成変化を測定することは患者自身が身体状態を把握するためには有意義である。

一方、被験者の一部費用(体力測定費用及び熟大メイト使用料、筋力測定費用、食事内容調査費、リブレpro費用)を当医局で負担することとし、これらのことを被験者に十分説明した上で、研究への参加の判断を求める。

## 18.3.　健康被害への対応と補償

本研究の実施に伴い、被験者に健康被害が発生した場合は、被験者の保険診療内で検査や治療など、適切な処置を講じる。

# 19. 研究期間と研究の終了・早期中止

## 19.1.　研究期間

症例登録期間：倫理委員会承認後～2019年12月31日

試験実施期間：倫理委員会承認後～2020年12月31日

## 19.2.　研究の終了

　最終登録被験者のデータ固定が終了した時点で本研究の終了とし、研究責任者は速やかに研究終了報告書を医学部長に提出する。

## 19.3.　研究の早期中止

　研究担当者は、以下の事項に該当する場合は、研究実施継続の可否を検討する。

1. 被験者の組み入れが困難で、予定症例数に達することが極めて困難であると判断されたとき
2. 予定症例数または予定期間に達する前に、研究の目的が達成されたとき
3. 本学倫理委員会により、実施計画等の変更の指示があり、これを受入れることが困難と判断されたとき

研究責任者は、本学倫理委員会により中止の勧告あるいは指示があった場合は、研究を中止する。また、研究の中止を決定した時は、速やかに医学部長にその理由とともに文書で報告する。

# 20. 医療機器等の保存及び使用方法並びに保存期間

該当せず

# 21. 記録の保存

本研究に係る試料・情報（研究データ等）は、管理責任者（研究責任者）のもと、試料は論文等の発表後５年間、情報（資料）は論文等の成果発表後10年間、当科の施錠可能な冷凍庫及び保管庫に保管する。保管期間終了後、試料は、匿名化されたまま密封容器に廃棄あるいは焼却処分する。紙データは匿名化されたままシュレッダー等を用いて廃棄し、電子データはデータを完全に消去する。

# 22. 研究の公表と成果の帰属

## 22.1.　研究計画の登録

　本研究は、UMIN 臨床試験登録システム（http://www.umin.ac.jp/ctr/index-j.htm）のデータベースへ臨床試験登録し、研究実施計画の変更および研究の進捗に応じて適宜更新し、研究を修了したときは遅滞なく当該試験の結果を登録する。

## 22.2.　成果の帰属

　本研究の成果は信州大学に帰属するものとする。研究責任者は、本研究の成果は関連学会での発表や論文を通じて公表する。

# 23. 研究実施体制

　本研究は、以下の体制で実施する。

【研究分担者】

○　信州大学医学部附属病院　糖尿病内分泌代謝内科 　 医員 北島　浩平

　　信州大学医学部　　　　　内科学第四教室 　　 教授　 　 駒津　光久

　　信州大学医学部　 内科学第四教室　　 　 准教授 　　 山崎　雅則

信州大学医学部附属病院　糖尿病内分泌代謝内科　　 助教 　佐藤　亜位

信州大学医学部附属病院　糖尿病内分泌代謝内科　 　助教　　 　　　　　　　 大岩　亜子

信州大学医学部附属病院　糖尿病内分泌代謝内科 　助教　　　　 　 　大久保　洋輔

信州大学医学部　 内科学第四教室　 助教　　　 　 柴田 有亮

信州大学医学部 　　　　　 内科学第四教室　 助教 　　 　　北原　順一郎

信州大学医学部附属病院　糖尿病内分泌代謝内科　 医員　　 　　 服部　由紀子

信州大学医学部附属病院　糖尿病内分泌代謝内科 　 医員 兼子　敦子

信州大学医学部附属病院　糖尿病内分泌代謝内科　 医員 中村　純子

信州大学医学部附属病院　糖尿病内分泌代謝内科　 医員 関戸　恵子

（○ 研究責任者）

【インターバル速歩の情報管理・体力測定責任者】

熟年体育大学リサーチセンター　　　　健康推進コーディネータ　　 　　　降幡 真由佳

【インターバル速歩におけるアドバイザー】

信州大学先鋭領域融合研究群バイオメディカル研究所　　教授 　　　　　 　増木　静江

信州大学大学院　医学科　加齢生物学　　　　　　　　　特任教授　　　 　　能勢　博

【画像検査情報責任者】

信州大学医学部　医学科　画像医学教室　　 　　　　　　教授　　　　　 藤永　康成

【個人情報管理者】

信州大学医学部附属病院 糖尿病内分泌代謝内科 医師事務作業補助者　　細川　眞奈美

【研究事務局】

信州大学医学部附属病院　糖尿病内分泌代謝内科　　医局　　0263‐37‐2686

【データマネジメント実施施設】

信州大学医学部附属病院臨床研究支援センター　データ管理グループ

【統計解析責任者】

株式会社　エスアールディ

【モニタリング実施施設】

　　信州大学医学部附属病院臨床研究支援センター　モニタリンググループ

# 24. 参考資料・参考文献

1) Tamura,Y. et al.「Effects of diet and exercise on muscle and liver intracellular lipid contents and insulin sensitivity in type2 diabetic patients.」.J clin Endocrinal Metab,90(2005):3191-3196.

2) R. D. Reid. Et.al.「Effects of aerobic exercise, resistance exercise or both,on patient-reported health status and well-being in type 2 diabetes mellitus: a randomised trial」Diabetologia (2010) 53:632–640

3) Kristian,K. et al「The effects of Free Living Interval Walking Training on glycemic control,body composition,and physical fitness in Type 2 diabetic patients」.Diabetic Care 36(2013):228-236.

4) Shuichi Handa et.al.「Target intensity and interval walking training in water to enhance physical fitness in middle-aged and older women: a randomised controlled study」Eur J Appl Physiol.23 september 2015

5)Ronald J Sigal et.al.「Effects of aerobic training, resistance training, or both,on glycemic control in type 2 diabetes.」Ann Intern Med. 2007;147:357-369.
